# A Case-Control Study on the Oxidative Balance of 50% Autologous Serum Eye Drops

**DOI:** 10.1155/2016/9780193

**Published:** 2016-08-21

**Authors:** Patrícia Ioschpe Gus, Diane Marinho, Samira Zelanis, Adriane Belló-Klein, Claudete Locatelli, Felipe Nicola, Ana Laura Kunzler, Tania Regina Gatelli Fernandes, Cristina Campos Carraro, Luciene Barbosa

**Affiliations:** ^1^Faculty of Medicine, Universidade Federal de São Paulo, 04023-062 São Paulo, SP, Brazil; ^2^Hospital de Clínicas de Porto Alegre, 90035-903 Porto Alegre, RS, Brazil; ^3^Faculty of Medicine, Universidade Federal do Rio Grande do Sul, 90035-190 Porto Alegre, RS, Brazil; ^4^Laboratory of Cardiovascular Physiology, Universidade Federal do Rio Grande do Sul, 90035-190 Porto Alegre, RS, Brazil; ^5^Department of Ophthalmology, Universidade Federal de São Paulo, 04023-062 São Paulo, SP, Brazil

## Abstract

*Importance.* Autologous serum (AS) eye drops are recommended for severe dry eye in patients with ocular surface disease. No description of the antioxidant balance of AS eye drops has been reported in the literature.* Objective.* This study sought to evaluate the total reactive antioxidant potential (TRAP) and concentration of reactive oxygen species (ROS) in samples of 50% AS eye drops and their correlations with the demographic characteristics and lifestyle habits of patients with ocular surface disease and healthy controls.* Design.* This was a case-control study with a 3-month follow-up period.* Participants*. 16 patients with severe dry eye disease of different etiologies and 17 healthy controls matched by age, gender, and race were included.* Results.* TRAP and ROS were detected at all evaluated times. There were no differences in the mean ROS (*p* = 0.429) or TRAP (*p* = 0.475) levels between cases and controls. No statistically significant differences in the concentrations of ROS or TRAPs were found at 0, 15, or 30 days (*p* for ROS = 0.087 and *p* for TRAP = 0.93). Neither the demographic characteristics nor the lifestyle habits were correlated with the oxidative balance of the 50% AS eye drops.* Conclusions and Relevance.* Both fresh and frozen 50% AS eye drops present antioxidant capacities and ROS in an apparently stable balance. Moreover, patients with ocular surface disease and normal controls produce equivalent AS eye drops in terms of oxidative properties.

## 1. Introduction

Dry eye is a common disorder of the tear film that affects millions of people over the age of 40 years worldwide, and there is currently no cure for this disease [1]. Artificial tears provide lubrication but lack the biologically active components found in natural tears, which have a complex composition that includes water, salts, hydrocarbons, proteins, and lipids. Additionally, the frequent application of artificial tear solutions containing chemical preservatives to prevent contamination has been found to induce toxic and allergic reactions, especially among those with sensitive eyes [2, 3].

Eye drops that are produced by separating the liquid and cellular components of a patient's blood have been demonstrated to possess many of the same biological nutrients that are found in natural tears [1, 4–6]. Serum and tears show similar constituent concentrations, with the exception of greater amounts of vitamin A, lysozyme, transforming growth factor-*β* (TGF-*β*), and fibronectin and reduced amounts of immunoglobulin A (IgA), epithelial growth factor (EGF), and vitamin C in serum compared with tears [1, 2, 4]. The use of autologous serum (AS) eye drops has been reported for the treatment of severe dry eye and ocular surface disorders, such as Sjögren's syndrome (SS), superior limbic keratoconjunctivitis, graft-versus-host disease, Stevens-Johnson syndrome, ocular cicatricial pemphigoid, recurrent corneal erosions, neurotrophic keratopathy, Mooren's ulcer, aniridic keratopathy, and postkeratorefractive surgery. AS eye drops are prepared as unpreserved diluted blood solutions [1, 7–9].

Due to its localization and function, the cornea is chronically exposed to ROS accumulation as well as to the oxidative stress; however, normal corneas have well-developed antioxidant defense systems which contain direct free radical scavengers such as glutathione peroxidase, superoxide dismutase, catalase, lactoferrin, and calcium [10–12]. In specific conditions, the increased ROS production and accumulation, the oxidative stress, and the prooxidant/antioxidant imbalance lead to the corneal pathologies, including ocular surface disease and dry eye syndrome [12–15]. Mitochondria-induced oxidative damage is strongly related to lacrimal gland inflammation and dysfunction, which was shown to result in dry eye disease in a conditional transgenic mouse model [16]. Increased oxidative stress in the conjunctivas of SS patients also appears to play a role in the pathogenesis of dry eye disease. Furthermore, the relationship between reactive oxygen species (ROS) production and lipid peroxidation-related membrane damage and inflammatory processes has been observed in dry eye patients with SS [10]. According to some studies, tobacco, UV radiation, photochemical stress, ozone, smoke, and exhaust gases can cause dry eye due to inflammation and ROS formation [17].

There is no information in the literature related to the oxidative balance of AS eye drops. However, knowledge regarding the antioxidant properties of AS eye drops might aid the understanding of the mechanism of action of AS eye drops in dry eye patients. This study measured the total reactive antioxidant potential (TRAP) and the concentrations of free radicals (ROS) in 50% AS eye drops and correlated the results with the demographic characteristics and lifestyle habits of patients with ocular surface disease and healthy controls.

## 2. Patients and Methods

Sixteen patients with ocular surface disease of different etiologies and 17 healthy controls matched for age and gender were enrolled to obtain 50% AS eye drops that were produced with their donated blood, which was acquired via venipuncture. 3 mL samples were generated from the 30 vials of AS eye drops that are typically produced monthly for each patient at the Ophthalmology Service of Hospital de Clínicas de Porto Alegre (HCPA). Additional 3 mL samples were produced from blood donated by controls and used for biochemical evaluation.

All patients and controls completed a questionnaire regarding demographic characteristics (e.g., age and gender), behavioral habits (smoking, alcohol, vegetables, fruit and grain intakes, exercise, and synthetic vitamin use), and medical diagnoses and signed an informed consent document that was approved by the Ethical Committee of HCPA.

*Preparation of the 50% AS Eye Drops.* The AS eye drops were prepared at the Molecular Analysis Unit of HCPA's Protein Research Center (UAMP) following the standards of the Infection Commission. Approximately 5 mL of peripheral venous blood was collected from the controls, and 150 mL was collected from the cases. The samples were centrifuged at 2,500 rpm for 10 minutes at room temperature 1 hour after venipuncture. The sera were removed from the tubes and added to 0.5% unpreserved methylcellulose at 1 : 1 dilution with laminar flow.

*Biochemical Evaluation.* Three Eppendorf vials containing 1.0 mL 50% AS eye drops from each patient and control were analyzed at the following 3 time points:

 Time 0: fresh AS eye drops; the biochemical evaluations were performed up to 48 hours after preparation.
 Time 1: frozen AS eye drops, maintained at −20°C for 15 days and then thawed for analysis.
 Time 2: frozen AS eye drops, maintained at −20°C for 30 days and then thawed for analysis.

The biochemical evaluations measured TRAP, which was predominantly determined by glutathione and ascorbic acid, and ROS, which were mainly composed of superoxide and hydrogen peroxide. Details of the evaluations are provided below.

TRAP represents the nonenzymatic antioxidant capacity of the tissue as determined by measuring the intensity of luminol (LH2) chemiluminescence induced by the addition of 2,2′-azobis(2-amidinopropane) (ABAP) at ambient temperature. This method is based on the oxidative induction time of lipid dispersion exposed to ABAP, which represents a source of free radicals with a constant and known production rate. For the basal chemiluminescence count, 4 *μ*L of 10 mM glycine buffer was added to the ABAP solution. Next, 10 *μ*L of 80 mM Trolox glycine buffer (water soluble vitamin E, which is the gold standard) or 10 *μ*L of tears (test sample) was added to the ABAP buffer solution. In both solutions, the chemiluminescence intensity due to LH2 is reduced as antioxidants are consumed. The time required for the count to return to the initial known value is proportional to the antioxidant capacity, and these times for the Trolox (vitamin E) and tear solutions were compared. This standard curve is constructed with three crescent concentrations of Trolox (in *μ*M). Antioxidant capacity of samples is calculated based on the standard curve, one Trolox unit being correspondent to 1 *μ*M, and the results are expressed as *μ*M. TRAP was measured in a beta counter (LKB Rack Beta Liquid Scintillation Spectrometer 1215, LKB-Produkter AB, Bromma, Sweden).

ROS generation was measured based on the fluorescence emission of dichlorofluorescein diacetate (DCF-DA) (Sigma-Aldrich, SL, USA). DCF-DA permeates the cell membrane and is rapidly oxidized by intracellular ROS to form the compound 2,7-dichlorofluorescein (DCF), which is highly fluorescent. The samples were excited at 488 nm, and the emission at 525 nm was collected. The results are expressed in nmol per mg of protein.

## 3. Statistical Analysis

The descriptive data were collected, and the means were compared using generalized linear models. Mann-Whitney and Kruskal-Wallis tests were used for the nonparametric analyses of independent samples. The number of samples was calculated to provide a power of 95%. The data were analyzed with IBM SPSS 22 statistical software.

## 4. Results

**Figure 1:**
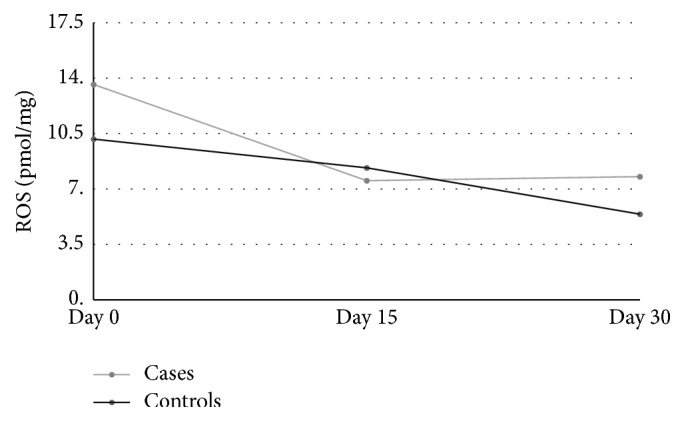
ROS concentration over time: cases × controls.

**Figure 2:**
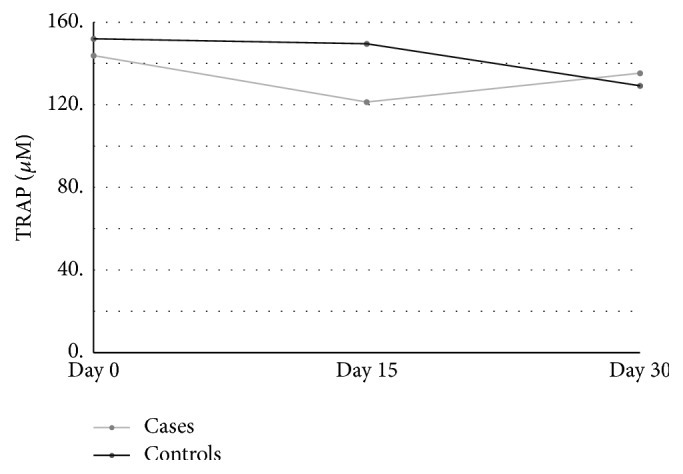
TRAP concentration over time: cases × controls.

A total of 33 subjects were included (16 cases and 17 controls). Forty-seven percent of the subjects were male. Questionnaire variables (Table 1) exhibited no relationships with the independent variables tested (Table 2). The TRAP and ROS concentrations were identified in all samples. There were no differences in the mean concentrations of ROS (*p* = 0.429) or TRAP (*p* = 0.475) between the cases and controls. There were no differences in the TRAP or ROS concentrations in the samples from each individual over time (*p* = 0.361). Furthermore, when all of the 3 ROS and TRAP values from the different time points were compared between groups, no differences were found (*p* for ROS = 0.087 and *p* for TRAP = 0.93; Table 3 and Figures 1 and 2).

## 5. Discussion

The Definition and Classification Subcommittee of the International Dry Eye Work Shop [18] redefined dry eye as a multifactorial disease of the tears and ocular surface which results in symptoms of discomfort (including foreign body sensations, dryness or irritation, burning, light sensitivity, and redness), visual disturbances, and tear film instability with potential damage to the ocular surface. Dry eye is accompanied by an increased osmolarity of the tear film and inflammation of the ocular surface. Recent studies have demonstrated that oxidative stress is involved in the pathogenesis of dry eye disease, keratoconus, bullous keratopathy, and Fuchs endothelial dystrophy [18–20]. In addition, higher levels of ROS, lipid oxidative stress markers, and inflammatory cells have been found in the conjunctiva and tear film of SS patients [10]. However, few studies have comprehensively investigated the relationship between hyperosmolarity and oxidative damage on the human ocular surface or the antioxidant properties of therapeutic preparations [21].

Ioschpe Gus et al. first reported the antioxidant properties of the tears of healthy individuals, which were acquired via lacrimal stimulation. The study by Ioschpe Gus et al. employed the same technique of TRAP detection as the present study [11]. Based on the results of both studies, 50% SA eye drops seem to contain TRAP concentrations that are approximately five to six times greater than TRAP in the natural tears of young and healthy individuals. Ioschpe Gus et al. did not measure ROS in tears.

The oxidative stress components and antioxidant properties seem to remain stable over time in both fresh and thawed samples. This finding is interesting because patients must use several vials of AS, as each vial is used for a maximum of approximately 48 h, and some of the last vials are thawed 30 days after preparation. Moreover, ROS stability allows the authors to infer that there is no microorganisms' contamination over time, since it would cause the rise of ROS in samples [22]. The authors could not find comparable studies about the concentration of ROS in tears.

Although many systemic diseases are oxidative stress-related, and lifestyle might influence the oxidative balance in general, no differences in the antioxidant properties of the AS eye drops made from the cases and controls in the present study were found. This finding is important because it demonstrates that patients with ocular disease with and without systemic pathologies might be capable of producing high-quality AS eye drops in terms of their antioxidant properties.

AS eye drops improve the quality of life of severe dry eye patients, although reports of the correlation between this improvement and clinical measurements are scarce in the literature. The lack of an agreed on standard dilution of serum for use in anterior segment applications has led to concentrations from 20% to undiluted serum reported in studies for dry eye disease or persistent epithelial defects [5]. Based on data reported in different studies, 20% AS eye drops are not associated with significant improvements in aqueous tear production as measured with Schirmer's test or improvements in the condition of the ocular surface as measured by fluorescein or Rose Bengal staining compared with preservative-free artificial tears [1]. Regarding tear film stability as measured by the tear breakup time (TBUT), only Kojima et al. (2005) found a clinically meaningful difference between 20% AS eye drops and artificial tears in participants with SS and non-SS dry eye after two weeks of treatment [23]. Other studies using 50% AS eye drops show improvement in Schirmer scores and effectiveness in treatment of persistent corneal epithelial defects [24, 25]. Fifty percent AS dilution with preservative-free methylcellulose 1 : 1 is routinely used at HCPA because the authors believe it provides better symptomatic relief. Moreover, the clinical evidence suggests that 50% AS eye drops produce dry eye disease improvements without the immune reaction that 100% AS eye drops might produce [1].

This is the first paper to present data regarding the oxidative balance of autologous serum eye drops and the behavior of this balance over time. In particular, our results demonstrated a difference between the antioxidant concentration of 50% AS eye drops and natural tears compared to the literature [11], which might explain the strong subjective relief patients report following the use of the 50% AS preparation. The authors strongly recommend further investigations comparing different concentrations of AS in terms of their antioxidant potential and ROS levels and objective clinical measurements in dry eye patients.

## Figures and Tables

**Table 1:** Questionnaire variables.

Demographic characteristics, behavioral habits, and medical diagnoses	Number of patients among cases (*N* = 16)	Number of patients among controls (*N* = 17)
*Mean age*	43,6	43,4
*Gender*		
Male	7	7
Female	9	10
*Smoking *	1	2
*Alcohol intake*	3	7
*Fruit intake *	15	15
*Vegetables intake *	10	15
*Cereals intake *	11	13
*Vitamin supplementation*	9	6
*Physical exercises *	7	13
*Systemic disease*		
Arterial hypertension	6	3
Rheumatic disease	4	1
Diabetes mellitus	0	0
Other	0	4

**Table 2:** Correlation between ROS (day 0) and TRAP (day 0) percentiles distribution versus demographic characteristics and lifestyle habits.

	Age	Smoke	Gender	Alcohol	Fruits	Vegetables	Cereals	Vitamins	Exercise	Systemic diseases
TRAP Day 0	*p* = 0.15	*p* = 0.38	*p* = 0.58	*p* = 0.31	*p* = 0.75	*p* = 0.27	*p* = 0.84	*p* = 0.45	*p* = 0.16	*p* = 0.77
ROS Day 0	*p* = 0.98	*p* = 0.95	*p* = 0.55	*p* = 0.10	*p* = 0.16	*p* = 0.32	*p* = 0.96	*p* = 0.97	*p* = 0.84	*p* = 0.81

**Table 3:** ROS and TRAP's means pairwise comparison between days 0, 15, and 30.

	ROS (pmol/mg)	TRAP (*μ*M)
Day 0 versus day 15	11.88 versus 7.93 (*p* = 0.815)	147.90 versus 135.44 (*p* = 0.47)
Day 0 versus day 30	11.88 versus 6.59 (*p* = 0.497)	147.90 versus 132.26 (*p* = 0.777)
Day 15 versus day 30	7,93 versus 6.59 (*p* = 0.194)	135.44 versus 132.26 (*p* = 1,000)
